# Seroprevalence and Risk Factors of Varicella Zoster Infection in Iranian Adolescents: A Multilevel Analysis; The CASPIAN-III Study

**DOI:** 10.1371/journal.pone.0158398

**Published:** 2016-06-29

**Authors:** Shervin Ghaffari Hoseini, Roya Kelishadi, Amir Kasaeian, Behrooz Ataei, Majid Yaran, Mohammad Esmaeil Motlagh, Ramin Heshmat, Gelayol Ardalan, Omid Safari, Mostafa Qorbani, Seyed Naseredin Mostafavi

**Affiliations:** 1 Infectious Diseases and Tropical Medicine Research Center, Isfahan University of Medical Sciences, Isfahan, Iran; 2 Pediatrics Department, Child Growth and Development Research Center, Research Institute for Primordial Prevention of Non-communicable Disease, Isfahan University of Medical Sciences, Isfahan, Iran; 3 Hematology-Oncology and Stem Cell Transplantation Research Center, Tehran University of Medical Sciences, Tehran, Iran; 4 Nosocomial Infection Research Center, Isfahan University of Medical Sciences, Isfahan, Iran; 5 Acquired Immunodeficiency Research Center, Isfahan University of Medical Sciences, Isfahan, Iran; 6 Pediatrics Department, Ahvaz Jundishapur University of Medical Sciences, Ahvaz, Iran; 7 Chronic Diseases Research Center, Endocrinology and Metabolism Population Sciences Institute, Tehran University of Medical Sciences, Tehran, Iran; 8 Pediatrics Department, Alborz University of Medical Sciences, Karaj, Iran; 9 Dietary Supplements and Probiotics Research Center, Alborz University of Medical Sciences, Karaj, Iran; 10 Endocrinology & Metabolism Research Center, Endocrinology and Metabolism Clinical Sciences Institute, Tehran University of Medical Sciences, Tehran, Iran; University of California Riverside, UNITED STATES

## Abstract

The objective of this study was to evaluate the *varicella zoster* virus (VZV) immunity in Iranian adolescents. It was conducted as a primary study for vaccine implementation, and to investigate the association of climatic and socioeconomic factors with the epidemiology of this infection. In this cross- sectional study, anti VZV antibodies were measured in serum samples obtained in a national school-based health survey (CASPIAN- III). Association of demographic, socio-economic, and climate of the living region with the frequency of VZV was investigated by multivariate multilevel analysis. Overall, sera of 2753 individuals aged 10–18 were tested for VZV antibodies, from those 87.4% were positive. The prevalence was statistically different in four socio-geographic regions (P<0.001), varying between 85.24% in West region (mostly mountainous areas with cold climate) to 94.59% in Southeast region (subtropical climate). Among variables studied, only age and mean daily temperature of the living area were positively associated with the VZV seroprevalence. Our findings show that most Iranians develop immunity to VZV before the age of 10, but a substantial proportion of them are yet susceptible to the infection. Therefore, it seems that the best strategy to reduce the burden of the disease is to vaccinate high- risk adults, i.e. those without a history of varicella infection. The regional temperature might be the only determinant of VZV epidemiology in Iran.

## Introduction

Chickenpox (varicella) is an extremely infectious viral disease with universal distribution, which is caused by *Varicella zoster* virus (VZV). Although varicella is typically a self-limited illness, the course may infrequently be complicated by secondary bacterial infections, transient cerebellar ataxia, and disfiguring scars. Life threatening events including pneumonia, necrotizing fasciitis, encephalitis, septicemia, and disseminated varicella rarely may be observed, as well [1]. Congenital varicella syndrome, which is contracted by transmission of VZV to fetus during pregnancy, is another worrying complication [2]. After primary VZV infection, the virus becomes dormant in sensory nerve ganglia. Then, following subsequent reactivation, this virus may produce zoster (shingles), a pruritic vesicular exanthema with local distribution in a dermatome. Zoster may occasionally cause permanent neurological impairments including post-herpetic neuralgia, cranial nerve palsies, and visual defects [1]. In general, complications and mortality of VZV infection are more commonly detected in immuno-deficient patients, and are more frequently observed in adults than in the pediatric population [3].

While VZV-specific immune globulin or antiviral medications can prevent or alleviate severe varicella infection and may reduce the risk of complications, the only way for controlling varicella in a community is extensive VZV immunization [3]. Documentation of safety, efficacy and cost-effectiveness of VZV vaccination was assessed in several studies, and introduced these vaccines into the routine vaccination schedules of some developed countries including the United States, some European countries, and Australia [4–6].

So far, varicella vaccine is not part of the Iranian national immunization program and its usage is not allowed in private clinics [7]. On the other hand, it can be combined with the measles, mumps, and rubella (MMR) vaccine, which is actually integrated in the routine vaccination program in Iran with coverage of more than 90% [7]. The World Health Organization (WHO) recommends that routine childhood immunization against varicella would be considered in countries where this infection poses relatively significant socioeconomic or health problems [3]. The susceptibility rate of adult population to varicella infection is a pertinent factor for deciding about the introduction of VZV vaccine in routine childhood immunization of the community.

Limited seroprevalence studies have been conducted in Iran about the susceptibility of adult population to varicella infection; most of them have been conducted in restricted areas of the country and have included specific groups such as health care workers, medical students, and premarital or pregnant women. Thus, such findings cannot serve as a representative sample of the whole population [8]. A systematic review on studies from Iran reported that more than 40% of adolescents aged 11–15 were susceptible to VZV infection [8]. In previous studies, the prevalence ranged from 27% in Kashan (center of Iran) [9] to more than 40% in the metropolitan Tehran [10] and Shiraz (south of Iran) [11].

Epidemiology of varicella is apparently different according to the climate: while in temperate regions, up to 90% of children are infected before the age of 10, only small proportions of children are seropositive in tropical and subtropical regions. Thus, the susceptibility to varicella is more common among adults living in tropical regions than in temperate climate [3]. Iran is a vast country with highly diverse climates varying from subtropical to temperate and cold mountainous environments [12], and with considerable socio-economic diversity. Therefore, it can be a good model for investigating environmental and social risk factors of VZV acquisition in a community.

The aim of this study was to evaluate the frequency of antibodies to VZV in a large population of Iranian adolescents living in different regions of the country, as a guiding data for policy making about vaccine implementation, and to investigate the effect of climatic and socio-economic factors on epidemiology of this infection.

## Materials and Methods

### Subjects and setting

In this multicenter cross-sectional study, serum samples obtained in a national health survey were analyzed. This national school-based health survey (CASPIAN-III study) was conducted in 2009–2010 on 10–18 year-old students. Participants were recruited through multistage, cluster sampling method from 27 provinces of Iran, as explained previously [13]. Eligible schools were stratified and randomly selected according to the information bank of Ministry of Education. In each province, stratification of schools was performed according to area of residence (urban/rural), and school grade (elementary/ intermediate/ high school) with equal sex ratios, and students were randomly selected. A questionnaire containing demographic and household socio-economic information was filled by one of the parents. Blood samples were obtained from students and tested at selected provincial laboratories and residual serum samples were stored at -70°C.

In the current study, residual samples from 20 provinces were tested at the laboratory of Infectious Diseases and Tropical Medicine Research Center, Isfahan University of Medical Sciences. Some samples including those from remaining seven provinces were either unsuitable for testing or unidentifiable because of missing codes and were excluded from the study.

Demographic data and related household risk factors were extracted from CASPIAN-III database. Using principle component analysis variables including parents’ education, parents’ job, possessing private car, school type (public/private), type of home (private/rented) and having personal computer at home were summarized in one main component named socio-economic status (SES) score. A lower score corresponds to a lower SES. The method and variables which was used for calculating SES of family was approved previously in the Progress in the International Reading Literacy Study (PIRLS) for Iran [14].

Regional information for each province such as population density (the ratio of population to area for each province) and budget (the ratio of income to costs for each family) were obtained from 2011 national census data [15]. Climatic parameters were extracted from databases of Iran Meteorological Organization [16]. Iran was divided into four sub-national regions according to a previous published study [17]. In order to determine sub-national regions, combination of two criteria (geography and SES) was used [17].

Total antibodies to VZV (IgM/ IgG) were measured by a commercial enzyme linked immunosorbent kit (Vircell, Granada, Spain) according to the kit instruction. The sensitivity and the specificity of the test were 98% and 97% respectively.

### Ethical statement

The CASPIAN-III survey was approved by the institutional review boards of the Ministry of Health and Medical Education and the Ministry of Education and Training, as well as the collaborating universities at provincial level. The current study was also approved by the ethical committee of the Alborz University of Medical Sciences (project number: 2015.65.786). Written informed consent and oral assent were obtained from parents and students, respectively.

### Statistical methods

Continuous and categorical variables are expressed as mean (SD) and number (percentage) respectively. Qualitative and quantitative variables across regions were assessed using Chi square and ANOVA test respectively. Considering the effect of mix of individual level and provincial level factors on the frequency of VZV, besides taking account of clustering (provinces and regions) in our study, we used hierarchical multilevel modeling. Generalized Linear Mixed Models were used to perform the univariate and multivariate multilevel analysis to evaluate association of VZV seroprevalence with independent variables. Individual- and provincial- level variables that were associated with VZV seroprevalence in univariate analysis (P<0.2) were successively included in a multivariable multilevel model. Results of univariate and multivariate multilevel analysis are presented as odds ratio (OR) and 95% confidence interval (CI). Statistical analysis was performed using R language (R Package “lme4”) [18, 19]. P-value<0.05 was considered statistically significant in all statistical models. All models were estimated via restricted maximum likelihood (REML) [20].

## Results

Overall, 2753 individuals aged 10–18 were tested for VZV antibodies, of them 2406 (87.40%) had positive test. The positive percentages for socio-geographical regions were 94.59% for Southeast, 89.94% for North-Northeast, 85.24% for West and 86.31% for Central region. Table 1 displays the distribution of the individual- and provincial-levels variables according to the four socio-geographical regions.

**Table 1:** Individual-and provincial-level characteristics of studied population by regions: the CASPIAN-III Study.

			Study regions			
Characteristics	Southeast n = 296	North-Northeast n = 537	West n = 1321	Central n = 599	National n = 2753	P-value ^a^
**Individual level n (%)**						
VZV (positive)	280 (94.59)	483 (89.94)	1,126 (85.24)	517 (86.31)	2406 (87.40)	<0.001
Sex (female)	133 (45.08)	270 (50.37)	657 (49.81)	297 (49.58)	1360 (49.3)	0.40
Living area (urban)	177 (59.8)	344 (64.06)	883 (66.84)	403 (67.28)	1810 (65.7)	0.008
Number of siblings ^b^						<0.001
1–2	95 (32.87)	221 (42.42)	413 (32.55)	218 (37.26)	949 (35.6)	
3–4	114 (39.45)	202 (38.77)	539 (42.47)	249 (42.56)	1105 (41.4)	
> = 5	80 (27.68)	98 (18.81)	317 (24.98)	118 (20.17)	613 (23)	
**Individual-level mean (95%CI)**						
Age (year)	14.40 (14.12;14.68)	14.39 (14.20;14.58)	14.40 (14.26;14.53)	14.34 (14.14;14.54)	14.38 (14.28;14.48)	0.65
SES (score)^c^	0.05 (-0.15; 0.25)	0.14 (-0.01–0.29)	-0.16 (-0.25;-0.06)	0.19 (0.05;0.33)	0.000(-0.064–0.066)	<0.001
**Province-level mean (95%CI)**						
Population density (N/m^2^)^d^	15.00 (5.88;24.12)	96.75(25.29;168.21)	61.44 (50.93;71.96)	248.25 (0.0;696.41)	98.9 (9.77;188.02)	0.074
Budget (ratio)^e^	0.97 (0.92;1.02)	1.09 (1.02;1.15)	0.89 (0.84;0.94)	0.94 (0.82;1.07)	0.95 (0.91;0.99)	0.006
Mean daily temperature (°C)	19.80 (12.26; 27.34)	15.38 (13.26; 17.49)	14.67 (11.24; 18.10)	16.70 (14.38; 19.02)	15.98 (13.94;18.03)	0.071
Humidity (percent)	44.00 (22.93; 65.07)	68.25 (53.8; 82.71)	50.67 (43.79; 57.54)	39.00 (32.55; 45.45)	50.85 (44.07;57.63)	0.024

VZV: Varicella zoster virus; SES: Socio-economic status

^a^ By Chi square and ANOVA test.

^b^ Missing values were excluded for calculating percentages.

^c^ The SES score summarizes different variables including: parents’ education, parents’ job, possessing private car, school type (public/private), type of home (private/rented) and having personal computer at home.

^d^ The ratio of population of a province to its area.

^e^ The ratio of household income to household costs.

Considering individual-level variables, the age and the sex had similar distribution across regions, while the number of children in a family, living area (urban/rural), and SES scores were statistically different between regions. Among provincial level covariates, budget and humidity were significantly heterogeneous across regions (p<0.05).

Table 2 presents the results of the univariate models. It shows that the age and the number of children in a family were associated with VZV seropositivity at 0.2 level of significance. Besides, mean daily temperature and budget were the provincial-level variables associated with VZV seropositivity at 0.2 level of significance.

**Table 2:** Association of independent variables at individual-and provincial-level with *Varicella zoster* seroprevalence in univariate multilevel model: the CASPIAN-III Study.

	Odds ratio	95% CI	Total	P-value ^a^
**Individual-level variables**				
Sex			2749	0.573
Male	0.94	0.75–1.17		
Female	1			
Area			2753	0.412
Urban	1.11	0.87–1.41		
Rural	1			
Number of siblings			2457	
**1–2**	0.77	0.56–1.05		0.102
**3–4**	0.83	0.61–1.13		0.228
**≥ 5**	1			
Age (year)	1.15	1.09–1.2	2753	<0.001
Socio-economic status (score)^b^	0.98	0.91–1.06	2664	0.680
**Province-level variables**				
Population density (N/m^2^)^c^	1.00	0.999–1.001	2753	0.594
Budget (ratio)^d^	4.94	0.79–30.99	2753	0.088
Mean daily temperature(°C)	1.06	1.004–1.11	2753	0.033
Humidity (percent)	1.01	0.99–1.03	2753	0.367

^a^ According to logistic regression test.

^b^ The Socio-economic status score summarizes different variables including: parents’ education, parents’ job, possessing private car, school type (public/private), type of home (private/rented) and having personal computer at home.

^c^ The ratio of population of a province to its area.

^d^ The ratio of household income to household costs.

Four significant variables of age, number of children in a family, mean daily temperature, and budget were obtained from univariate models; including them in a final model showed that one individual level variable (age, per years: OR: 1.15, 95% CI: 1.09–1.2) and one provincial-level variable (mean daily temperature, per degree centigrade: OR: 1.06, 95%CI: 1.004–1.11) were associated with the VZV seropositivity at the 0.05 level of significance (Table 3).

**Table 3:** Association of independent variables at individual- and provincial-level with *Varicella zoster* seroprevalence in multivariate multilevel model: the CASPIAN-III Study.

Independent variables	OR (95% CI)	P-value ^a^
Age (year)	1.14 (1.089–1.2)	<0.001
Number of children		
1–2	0.95 (0.68–1.32)	0.764
3–4	0.91 (0.67–1.25)	0.563
≥ 5	1	
Mean daily temperature(°C)	1.06 (1.01–1.12)	0.045
Budget (ratio)^b^	2.42 (0.36–16.10)	0.360
2664 individuals; 4 regions; 20 provinces		

^a^ According to logistic regression test.

^b^ The ratio of household income to household costs.

**Fig 1:**
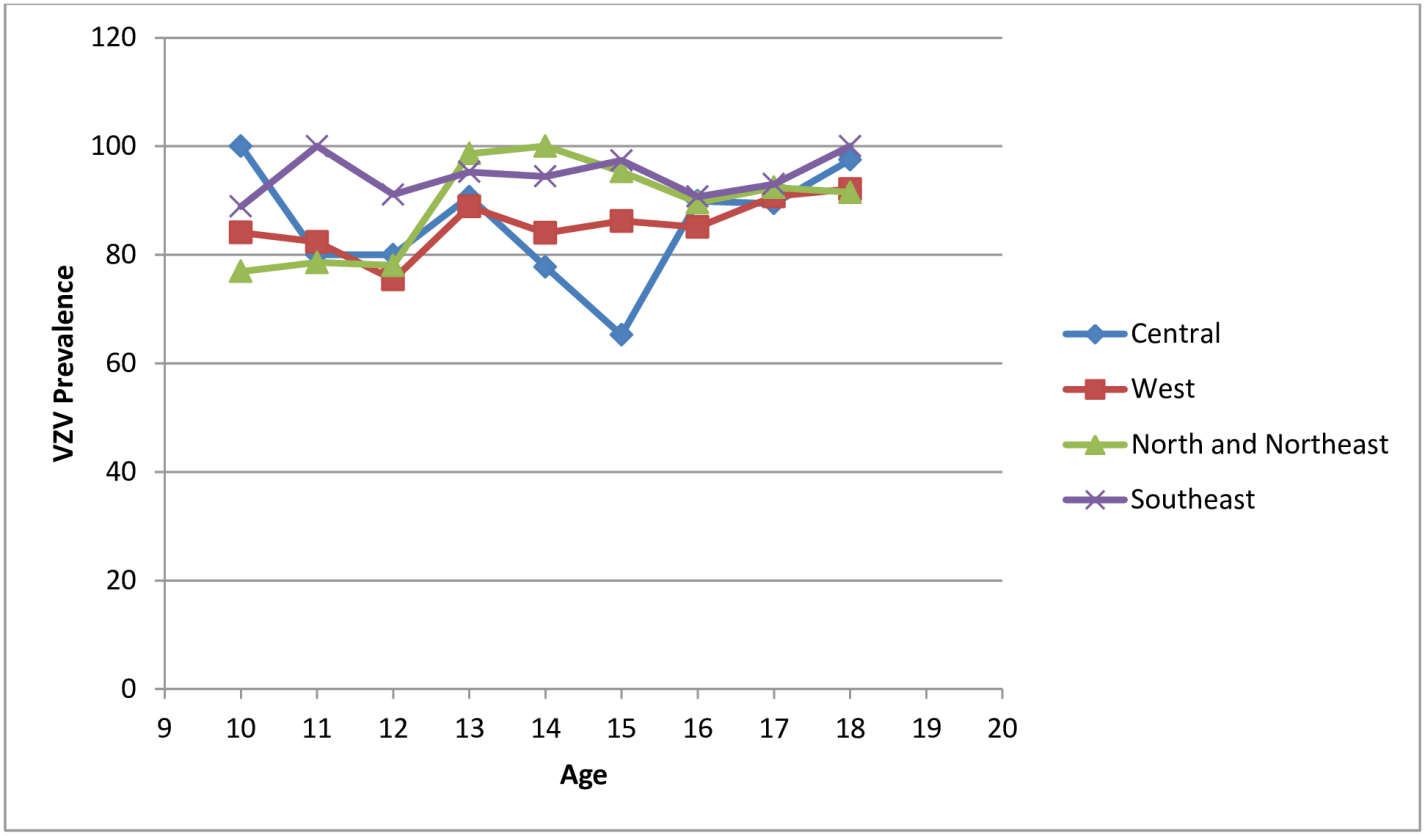
*Varicella zoster* age specific seroprevalence in four socio-geographic regions of Iran: the CASPIAN-III Study.

**Fig 2:**
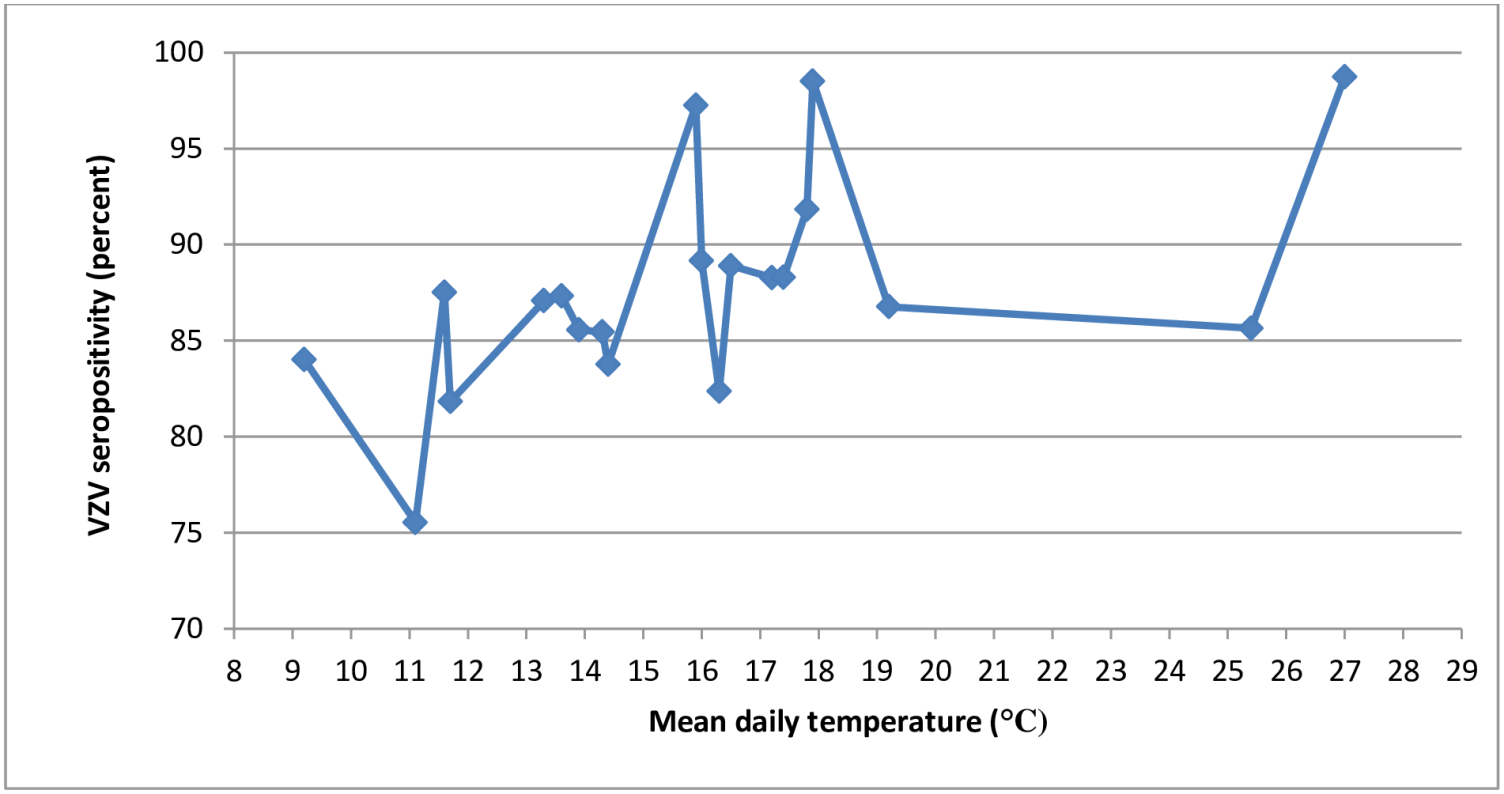
Association of *Varicella zoster* seroprevalence with mean daily temperature in 20 provinces of Iran: the CASPIAN-III Study.

Table 4 represents the age specific prevalence of VZV antibodies. Figs 1 and 2 show the association of age with seroprevalence of VZV in four socio-geographic regions and the association of mean daily temperature with seroprevalence of VZV in 20 provinces of Iran respectively.

**Table 4:** *Varicella zoster* age specific seroprevalence in Iranian adolescents: the CASPIAN-III Study.

Age	Positive VZV antibody/ total (percent)	95% CI
10	71/ 82 (86.58)	79.05–94.12
11	249/ 299 (83.28)	79.02–87.53
12	382/ 485 (78.76)	75.11–82.42
13	296/ 322 (91.92)	88.93–94.92
14	214/ 247 (86.63)	82.37–90.91
15	137/ 152 (90.13)	85.34–94.93
16	365/ 417 (87.53)	84.35–90.71
17	375/ 412 (91.01)	88.25–93.79
18	317/ 337 (94.06)	91.53–96.60

VZV: *Varicella zoster* virus

## Discussion

In this study, the overall seroprevalence of VZV in 10-18-year-old adolescents was 87.40%, ranging from 85% to 95% in different socio-geographic regions, and from 65% to 100% in different ages.

This prevalence is much higher than some previous reports from Iranian teenagers. In a systematic review conducted on reports from Iran, VZV prevalence was reported as 59.44% (95% CI, 46.07–72.81) in 11-15-year-old children and 75.93% (95% CI, 70.20–81.66) in 16-20- year-old adolescents of nine provinces of Iran [8]. Our results are consistent with the previous studies conducted in 16-20- year-old population of Tehran in 2003–2005, which was reported as 87.5%, [21] and the study from Shiraz (South of Iran) in 2008 (83.2%) [11]. The frequency obtained in our study is higher than previous reports from Bushehr (South of Iran)[22] and Tehran [23] in year 2008, which were reported as 74% and 74.2%, respectively.

Although in our study, significant difference was documented in the VZV seroprevalence according to the region and age groups, but the ratios were not so divergent; the findings indicate that at age of 10, about 20% of Iranian children are susceptible to this infection, and at age of 18, the susceptibility declines to less than 10% in all four socio-geographic regions. It seems that the non-representative method of sampling or inadequate number of participants in previous studies can explain the differences of the current findings with some of the previous studies in Iran.

Our study revealed that although varicella is a childhood disease in Iran, but a substantial proportion of adolescents, i.e. about 20%, are at risk of developing this infection. VZV vaccination may abolish severe form of this disease in immune deficient patients, and may reduce complications as secondary bacterial infections and neurologic complications in normal children. Though, there are many concerns about introducing routine childhood vaccination at national level, particularly because there is no estimate of public health impact and socio-economic burden of the infection and cost effectiveness of universal vaccination, which need to be provided by a national surveillance system [3].

In this situation, it seems that the best strategy to control the VZV infection is to offer the vaccine to individual adolescents and adults without a history of varicella, in particular to at- risk population including women of childbearing age, health care providers, day-care personnel, teachers, college students, and military personnel. This recommendation is in accordance to the WHO statement for VZV immunization in developing countries, which prefer the introduction of other new vaccines with much greater public health impact, such as rotavirus, and pneumococcal vaccines, prior to introducing varicella vaccine in routine childhood immunization programs [3].

Iran is placed in the temperate zone of climate with the southern regions extending to the subtropical area and generally with an arid climate. Our results are roughly in agreement with the pattern of VZV prevalence in temperate regions such as Europe, North America, and Japan in pre-vaccination era [24–26]. An investigation in 11 European countries showed that the rate of susceptibility to VZV was about 1.2% to 10.8% in all ages from 10 to 19; an exception was Italy with a susceptibility rate of about 18% in the same age group [25].

Limited information is available about prevalence of VZV in countries neighboring Iran. In Pakistan, a tropical pattern of VZV epidemiology was found with a seroprevalence of 42.5% in the 11-15- year age group and 46.7% in the 16-20- year age group [27]. Our findings are consistent with two studies in Turkey; in one of them18.8% of adolescents and 11.7% of young adults were seronegative for VZV [28], and in the other one, 94.3% of individuals older than 15 were seropositive for VZV [29].

In our study, the Southeast region, with the subtropical climate, had the highest VZV seroprevalence, but because of diverse climates existing adjacent throughout the country, it is difficult to assign a specific climate to the mentioned socio-geographic regions. We found that regardless of age, the only risk factor associated with VZV seropositivity was the annual mean daily temperature: higher temperatures were associated with higher rates of seropositivity.

Meteorological factors have been identified as the main determinants of VZV epidemiology in many parts of the world. Temperature might affect survival, transmission, and also the severity of presentation of VZV infection (mild or severe disease), and the effect of temperature might be different in tropical and temperate regions. Generally, it is known that the incidence and prevalence of VZV primary infection is higher in temperate regions than tropics [30, 31]. Several studies in tropical countries also showed a higher rate of VZV infection in cooler regions, as well as an increase of its incidence in cooler seasons. For instance, VZV seroprevalence was higher in cooler states than in warmer states of Thailand and Taiwan [32, 33], lower humidity in cool seasons were associated with more VZV pediatric hospitalization in Hong Kong [34], and increase in the mean daily temperature was associated with decrease in outpatient visits for varicella in Shanghai, China [35]. This would be because of the susceptibility of VZV to high temperatures, as it is proposed that high temperature and humidity negatively affects transmission of VZV in tropics [36]. However, a different pattern of seasonality is seen in temperate regions with peaks of infection transmission occurring in spring rather than in cool months [30]. Studies on association of climatic factors with VZV epidemiology in temperate regions are limited. In a survey in Greece, hospitalization due to VZV was increased during summer time and decreased during autumn, but this increase was directly associated with wind speed rather than higher temperature in those time periods [30]. Findings of the present study indicate that the higher temperature favors the transmission of the virus in our region. The reason for this variation needs to be determined. It is proposed that the mode of transmission of airborne viruses is mainly via aerosols in temperate climate, but direct contact is more important in tropics [37]. High temperature and humidity in tropics may prevent its transmission via aerosols, but does not affect or may facilitate transmission via direct contact [38, 39]. We suppose that the main mode of VZV transmission might be direct contact in our region, which may explain the association of VZV seropositivity with higher temperatures. More detailed surveys are needed to determine the exact effect of meteorological factors on VZV epidemiology in temperate regions.

We found that gender and socio-economic factors including living area (rural/urban), ratio of income to costs, population density, number of household siblings, as well as parental literacy and job were not important risk factors for VZV infection in Iranian adolescents. Higher age group was related to increased seropositivity; however some discrepancy existed in the age- specific prevalence of VZV in different regions. This might be due to limited outbreaks in previous years or because of small sample size of some age-region subgroups.

Having five or more siblings in the household and lower household budget were related to higher VZV seropositivity, but differences were not significant in multivariate model. Household size has been considered as a risk factor for VZV transmission in tropical areas like Guinea Bissau with an extremely large household sizes (mean 14.5 people) [40]. But in areas with lower mean household sizes, attendance at childcare and school was documented as a much more important factor [25]. Mean household size In Iran is reported as 3.5 at year 2011, ranging from 3.2 in Gilan (North of Iran) to 4.3 in Sistan and Baloochestan (Southeast), which is parallel to the number of siblings [15]. This limited range may stand for insignificant correlation between this factor and VZV seropositivity in the current study. Doubling of VZV prevalence ratios in the age group of 6–10 relative to age group 11–15, which was observed in prior studies, confirm the role of schooling on VZV epidemiology in Iran [9, 10].

Study limitations: The first limitation is that we were not able to include all age groups in our study, thus we cannot provide a complete picture of VZV epidemiology in Iran. Likewise, data from a number of provinces were not available for this study. However, this survey is the first representative nationwide seroepidemiological study about VZV in Iran and also in the Middle East and North Africa (MENA) region; studied provinces were from different socio-geographic regions, covering more than 80% of the total adolescent population. For compensating the likely occurring bias because of depletion of samples of some provinces, we assessed pooled data of four socio-geographic regions for determining risk factors of VZV infection across the country.

Conclusion: Natural VZV infection immunizes most of Iranian children before the age of 10, but a substantial proportion of adults and adolescents of the country are susceptible to the infection and are at the risk of developing severe and complicated infections, as well as congenital varicella syndrome, which might have serious health effects. Therefore, it seems that the best strategy for controlling the infection is to offer the vaccine to those adolescents and adults who do not have a history of varicella, in particular to those at increased risk of contracting or spreading the infection.
